# Laparoscopic Repair Following a Delayed Presentation of Traumatic Diaphragmatic Hernia: A Case Report

**DOI:** 10.7759/cureus.57079

**Published:** 2024-03-27

**Authors:** Yuto Kitano, Koji Okamoto, Maki Ohnishi, Tatsuya Aoki, Kazushige Shibahara

**Affiliations:** 1 Department of Surgery, Toyama Red Cross Hospital, Toyama, JPN; 2 Department of Anaesthesiology, Toyama Red Cross Hospital, Toyama, JPN

**Keywords:** laparoscopic surgery, diaphragmatic injury, one-lung ventilation, traumatic diaphragmatic hernia (tdh), trauma

## Abstract

Traumatic diaphragmatic hernia is a rare condition that occurs after trauma, and some patients have a delayed presentation. A laparoscopic approach is rarely used to repair traumatic diaphragmatic hernias. We encountered a case of asymptomatic diaphragmatic hernia diagnosed after a comprehensive medical examination. A 71-year-old woman was diagnosed with a delayed presentation of traumatic diaphragmatic hernia with prolapse of the greater omentum owing to a traffic injury 20 years ago. Surgery was performed laparoscopically using three ports, and intraoperative respiratory management was performed using a double-lumen tube. The 2.5-cm-diameter hernial orifice was sutured under contralateral one-lung ventilation after the greater omentum was returned to the abdominal cavity. The patient's postoperative course was uneventful, and she was discharged on the third day. Intraoperative strategies such as respiratory management and the laparoscopic approach play a crucial role in ensuring favorable postoperative outcomes. The last follow-up was at six months post-operation, and the patient was doing well.

## Introduction

A diaphragmatic hernia is a prolapse of the abdominal organs into the thoracic cavity through a defect in the diaphragm that occurs congenitally in the neonate or is acquired secondary to trauma. Traumatic diaphragmatic hernia (TDH) is a relatively rare condition that occurs following thoracic or abdominal trauma, occurring in 0.46-1.6% of blunt trauma injuries [1,2]. A TDH usually develops acutely after trauma; however, some cases develop later in life. The diaphragm is composed of muscles extending from the sternum, ribs, and lumbar vertebrae and is fused at the diaphragmatic central tendon; thus, TDHs owing to blunt trauma injuries occur more frequently in the central tendon [3,4]. Surgical repair is the standard treatment for TDH. TDHs are traditionally treated by laparotomy or thoracotomy, with few reports on laparoscopic surgery. Laparoscopic surgery with abdominal insufflation tends to be avoided in TDH surgery owing to its effect on respiratory dynamics; however, laparoscopic surgery is considered in some patients with acute or delayed presentation of TDH if their general condition permits [5]. Laparotomy or thoracotomy should be performed in patients with unstable respiratory or hemodynamic conditions. In this study, we report the case of a 71-year-old woman with a history of traffic injury 20 years prior who was incidentally diagnosed with a left diaphragmatic hernia. The patient was diagnosed with delayed TDH based on the history of trauma and underwent laparoscopic surgery. Herein, we achieved good results with this surgery.

## Case presentation

**Figure 1:**
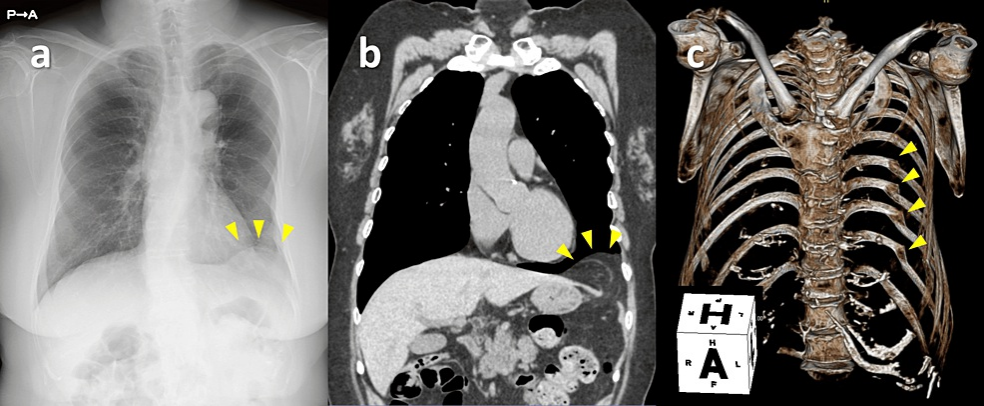
Imaging examinations. a) Chest radiography revealed a sharply demarcated soft tissue shadow on the left diaphragm. b) Computed tomography identified a diaphragmatic defect of approximately 2 cm and a protrusion of the greater omentum into the thoracic cavity. c) Healed fractures of the left eighth to 11th ribs were identified.

A 71-year-old woman was referred to our hospital for a detailed examination of an abnormal shadow in the left lung field, which was identified by chest radiography during a comprehensive medical examination to check her health condition. Radiography revealed a sharply demarcated soft tissue shadow on the left diaphragm (Figure 1a). Computed tomography revealed a left diaphragmatic hernia with a prolapsed greater omentum and healed multiple left rib fractures (Figure 1b, 1c).

After being referred to the Department of Surgery, a detailed medical history was reviewed, and she was diagnosed with delayed TDH owing to a traffic injury 20 years ago. The patient was hit by a car while riding a bicycle and injured her left back. Computed tomography showed multiple left rib fractures that were treated conservatively. There were no injuries to the thoracoabdominal organs other than rib fractures, including a diaphragmatic hernia at the time of injury. Except for a traffic injury, her medical history did not contribute to the development of a diaphragmatic hernia. Although she did not present with any subjective symptoms, there were concerns regarding the possibility of hernia enlargement and organ incarceration, and we decided to repair the hernia.

**Figure 2:**
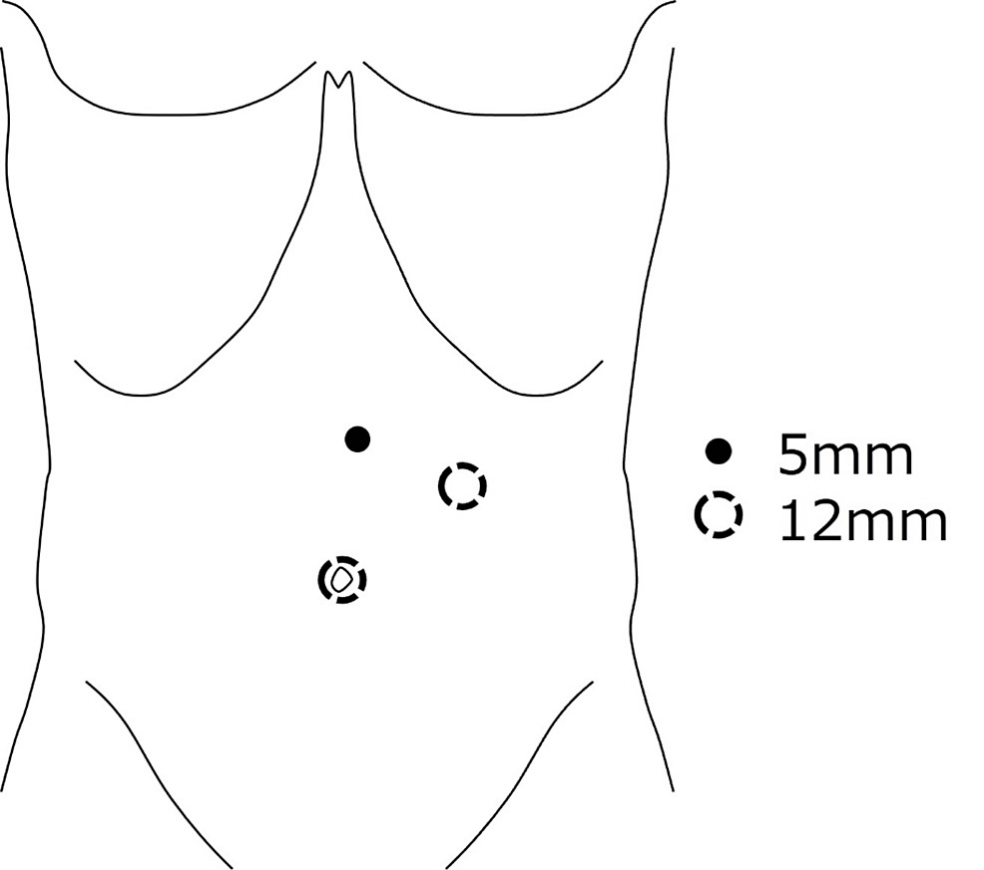
Port placement of the laparoscopic surgery. The surgery was performed laparoscopically with three ports. After the insertion of the first port for the scope at the umbilicus, two additional ports were added to the surgeon's forceps to form an isosceles triangle with the hernia site as the vertex while avoiding interference with the scope.

Laparoscopic surgery was performed under general anesthesia. A double-lumen tube was introduced in preparation for one-lung ventilation. Three laparoscopic ports were inserted, one for the scope and two for the surgeon's forceps (Figure 2).

**Figure 3:**
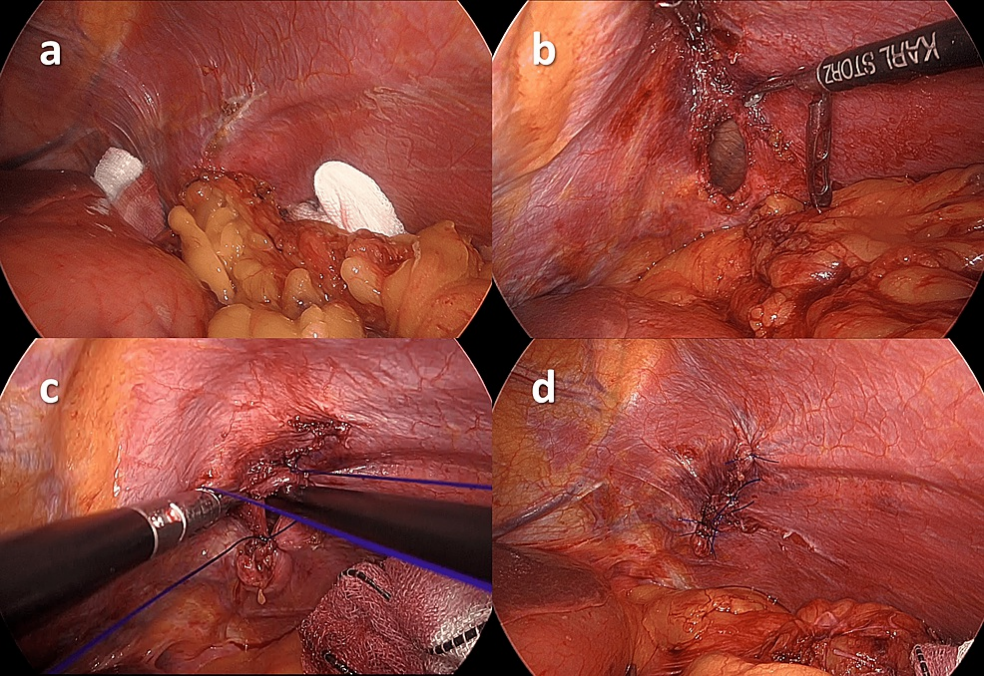
Intraoperative findings. a) The hernial orifice and greater omentum prolapsed into the left thoracic cavity were identified. b) The hernial orifice was oval with a longitudinal diameter of approximately 2.5 cm. c) The left thoracic cavity was suctioned just before the final suture. d) The hernia was closed with a non-absorbable monofilament thread.

Insufflation of the abdominal cavity was performed at a low pressure of 5 mmHg to avoid increased intrathoracic pressure. The left diaphragm that was adhered to the greater omentum was released, and the hernial orifice and greater omentum prolapsed into the left thoracic cavity (Figure 3a). The greater omentum was cautiously returned to the abdominal cavity to prevent hemorrhage, and the hernial orifice was completely exposed. A hernia was identified at the boundary between the tendinous and muscular parts of the diaphragm, corresponding to the American Association for the Surgery of Trauma (AAST) Classification of diaphragmatic injury grade 3. The intraoperative diagnosis was consistent with a delayed presentation of TDH because no findings suggestive of other congenital diaphragmatic hernias (the foramen of Bochdalek, Larrey, or Morgagni) were identified. The hernial sac (HS) was not identified, and the abdominal and thoracic cavities were contiguous. The hernial orifice was oval, with a longitudinal diameter of approximately 2.5 cm (Figure 3b). The orifice was closed with four-knotted sutures using a 3-0 non-absorbable thread without excessive tension. During the suturing of the diaphragm, right one-lung ventilation was performed to avoid lung injury and reduce diaphragmatic movement. The anesthesiologist pressurized the lungs, and the surgeon suctioned the thoracic cavity; immediately before the final suture threading, the diaphragm was ligated, interrupting the communication between the thoracic and abdominal cavities (Figure 3c). After the closure of the diaphragmatic hernia (Figure 3d), hemostasis of the returned omentum was confirmed, and a drainage tube for the surveillance of bleeding from the reduced omentum was placed under the left diaphragm through one of the laparoscopic ports to complete the surgery.

**Figure 4:**
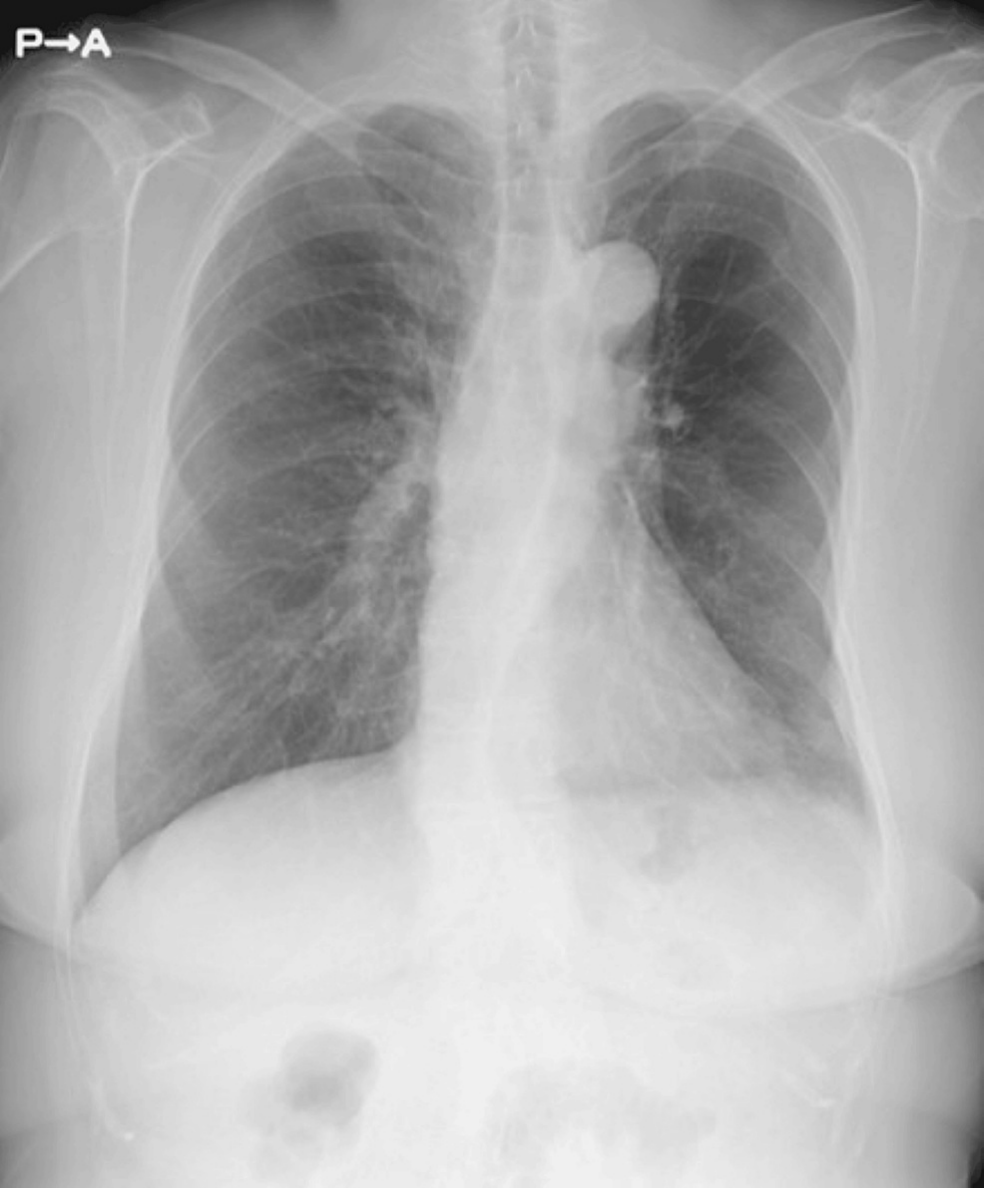
A chest radiography finding after discharge. The left diaphragm appeared smooth.

Thoracic drainage tubes were not required. The operative time was 76 min with minimal blood loss. The postoperative course was uneventful. The drain was removed on the second day, and the patient was discharged on the third day. Six months have passed since the surgery without recurrence (Figure 4).

## Discussion

TDHs occur in 0.46-1.6% of blunt trauma injuries [1,2]. Some cases of TDHs develop late, with reported rates ranging from 11.8% to 40% of all TDHs [6]. They occur more frequently on the left side because the right side is usually protected by the liver [7]. TDHs owing to blunt injury are more likely to occur at the central tendon disruption or at the boundary between the tendinous and muscular parts of the diaphragm, involving the physiological vulnerability of the diaphragm [3,4]. There appear to be regional differences in the type of trauma, with some reports indicating that penetrating trauma such as stab and gunshot wounds is the predominant cause of diaphragmatic hernia [2,8]. The AAST defines an Organ Injury Scale that classifies the degree of diaphragmatic injury into five different grades. The present case involved a laceration of the diaphragm that exceeded 2 cm, corresponding to grade 3 [9].

Clinical presentations of TDH are variable and may be asymptomatic or present with acute symptoms such as shortness of breath, shoulder pain, epigastric pain, and vomiting. Prolapsed intestines can cause intestinal obstruction, strangulation, or perforation, resulting in serious conditions [10,11].

TDHs are traditionally treated by laparotomy, thoracotomy, or both; however, laparoscopic surgery was recently reported in some cases. Transabdominal approaches including laparoscopic surgery are effective in repairing or resecting prolapsed organs. The transthoracic approach alone is often difficult to perform for prolapsed organs. The choice of approach largely depends on the skills of the surgeons involved because laparoscopic surgery requires more careful and gentler manipulation. Even if laparoscopic repair of diaphragmatic hernias and prolapsed organs cannot be completed, laparoscopic observation provides valuable information for the surgeon in the detailed assessment of the hernia site and prolapsed intra-abdominal organs and is recommended in the guidelines of the Eastern Association for the Surgery of Trauma [5]. Horton et al. reported that laparoscopic surgery was associated with fewer complications and shorter hospital stays than laparotomy, thoracotomy, or thoracoscopic surgery for Morgagni hernia, which is a type of diaphragmatic hernia [12]. The laparoscopic approach is superior in several aspects, including the visibility of the entire abdominal cavity and lesions. Furthermore, it can detect adverse events such as bleeding, organ damage, and intestinal ischemia and requires smaller incisions and faster postoperative recovery [13]. However, the patient should immediately undergo laparotomy or thoracotomy in cases of severe adhesions around the hernia and difficulty in returning the hernia contents or in cases of gastrointestinal tract injury or uncontrollable bleeding [7].

Most diaphragmatic hernias can be closed by direct suturing; however, a mesh should be used for repair in cases where the defects are large and cannot be closed. In a hernia with larger defects (>3 cm), an attempt to primarily repair the defect could lead to excessive tension owing to tissue loss and a high recurrence rate of 42%; therefore, a mesh should be used to reinforce the suture repair [14]. Considering the pressure gradient between the abdominal and thoracic cavities, mesh placement in the abdominal cavity is recommended [15]. When suturing a diaphragmatic hernia for closure, non-absorbable threads are preferred over absorbable threads to avoid recurrence [16].

Most TDHs are associated with abdominal and thoracic cavity communication during laparoscopic surgery owing to the lack of an HS. The intraoperative placement of a thoracic drain under laparoscopic control is effective in preventing postoperative pneumothorax [6]. However, one-lung ventilation on the opposite side with a double-lumen tube when suturing the diaphragm is effective in suppressing the movement of the diaphragm [17]. The authors also reported that intrapleural air could be eliminated by pressurizing the lungs as much as possible immediately before the complete closure of the diaphragmatic hernia, thereby eliminating the need for a thoracic decompression tube [17].

In this study, we encountered a rare case of delayed diaphragmatic hernia that was incidentally detected during medical examination. The patient was asymptomatic and underwent elective laparoscopic surgery for detailed observation and minimally invasive procedures. The use of a double-lumen tube allowed safe laparoscopic suturing of the diaphragm, and aspiration of the thoracic cavity immediately before the final closure of the hernial orifice eliminated the need for a thoracic tube. Non-absorbable simple sutures were used to prevent recurrence. A mesh was not used because the hernial orifice was not large and could be closed without excessive tension.

## Conclusions

We performed laparoscopic repair of delayed TDH with favorable outcomes. Laparoscopic surgery for TDH is effective to visualize the hernia site and evaluate the prolapsed organ, as long as the patient's general condition permits.

TDHs often have a defective HS and are associated with intraoperative communication between the abdominal and thoracic cavities. Placement of a thoracic drain may be avoided by using a double-lumen tube to ventilate one lung on the intact side and by suctioning the thoracic cavity before the closure of the diaphragmatic hernia.
